# Successful treatment of bilateral multiple pulmonary arteriovenous malformations in a patient with brain abscess and severe hypoxemia using a combination of transcatheter embolotherapy and surgical resection: a case report

**DOI:** 10.1186/s13019-021-01644-2

**Published:** 2021-09-08

**Authors:** Takahiro Ochi, Masako Chiyo, Takamasa Ito, Hideharu Furumoto, Toshihiko Sugiura, Yukio Saitoh

**Affiliations:** 1 Department of Thoracic Surgery, National Hospital Organization Chiba Medical Center, 4-1-2, Tsubakimori, Chuo-ku, Chiba 260-8606 Japan; 2 Department of Neurology, National Hospital Organization Chiba Medical Center, 4-1-2, Tsubakimori, Chuo-ku, Chiba 260-8606 Japan; 3 Department of Respirology Medicine, Chiba University Graduate School of Medicine, 1-8-1, Inohana, Chuo-ku, Chiba 260-8670 Japan

**Keywords:** Pulmonary arteriovenous malformation (PAVM), Hereditary hemorrhagic telangiectasia (HHT), Surgical treatment, Transcatheter embolotherapy, Brain abscess

## Abstract

**Background:**

A pulmonary arteriovenous malformation is an abnormal dilated blood vessel that makes direct communication between a pulmonary artery and pulmonary vein and can be associated with hypoxemia or neurological complications, including brain abscess and cerebral infarction. Treatment of pulmonary arteriovenous malformation includes surgical resection and transcatheter embolotherapy, however the adaptation of therapies should be considered when a patient is in bad condition.

**Case presentation:**

A 51-year-old man was admitted after developing fever, consciousness disorder, and hypoxemia. Magnetic resonance imaging of the brain showed a brain abscess. Bilateral pulmonary arteriovenous malformations were found by contrast computed tomography. Because of a family history of pulmonary arteriovenous malformation, a history of epistaxis, and the existence of oral mucosa telangiectasia, he was diagnosed with hereditary hemorrhagic telangiectasia and brain abscess caused by intrapulmonary right-to-left shunt. The brain abscess improved with antibiotic treatment; however, the administration of oxygen did not ameliorate his hypoxemia. His hypoxemia was exacerbated by positive pressure ventilation. Considering his systemic and respiratory condition, we considered surgery to involve a high degree of risk. After controlling his brain abscess and pneumonia, transcatheter embolotherapy was performed. This improved his systemic condition, enabling surgical treatment.

**Conclusions:**

This middle-aged patient suffering from brain abscess and severe hypoxemia with multiple pulmonary arteriovenous malformations was successfully treated by a combination of transcatheter embolotherapy and surgery. The adaptation and combination of therapies, as well as the sequence of treatments, should be considered depending on the patient status and lesions.

## Introduction

A pulmonary arteriovenous malformation (PAVM) is an abnormal dilated blood vessel that makes direct communication between a pulmonary artery and pulmonary vein. PAVMs are mainly found in patients with hereditary hemorrhagic telangiectasis (HHT) [1]. Because direct capillary-free communication results in anatomic right-to-left shunt, PAVM can be associated with hypoxemia or neurological complications, including brain abscess and cerebral infarction [1–3]. This report describes the case of a middle-aged patient suffering from a brain abscess, in whom multiple PAVMs were treated by the combination of transcatheter embolotherapy and surgical treatment, which achieved a radical cure that ameliorated hypoxemia and prevented the relapse of neurological complications, after the improvement of the brain abscess by antibiotic treatment.

## Case report

**Fig. 1:**
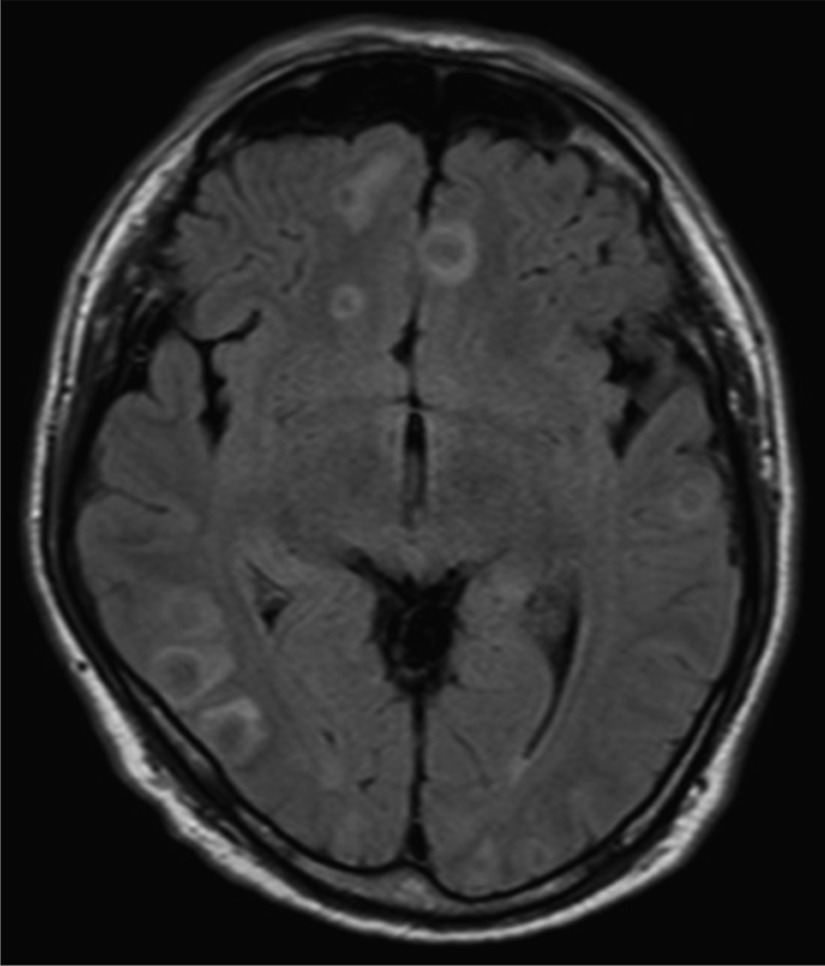
Enhanced head magnetic resonance imaging revealed multiple ring-enhancing lesions over the cerebrum and cerebellum

**Fig. 2:**
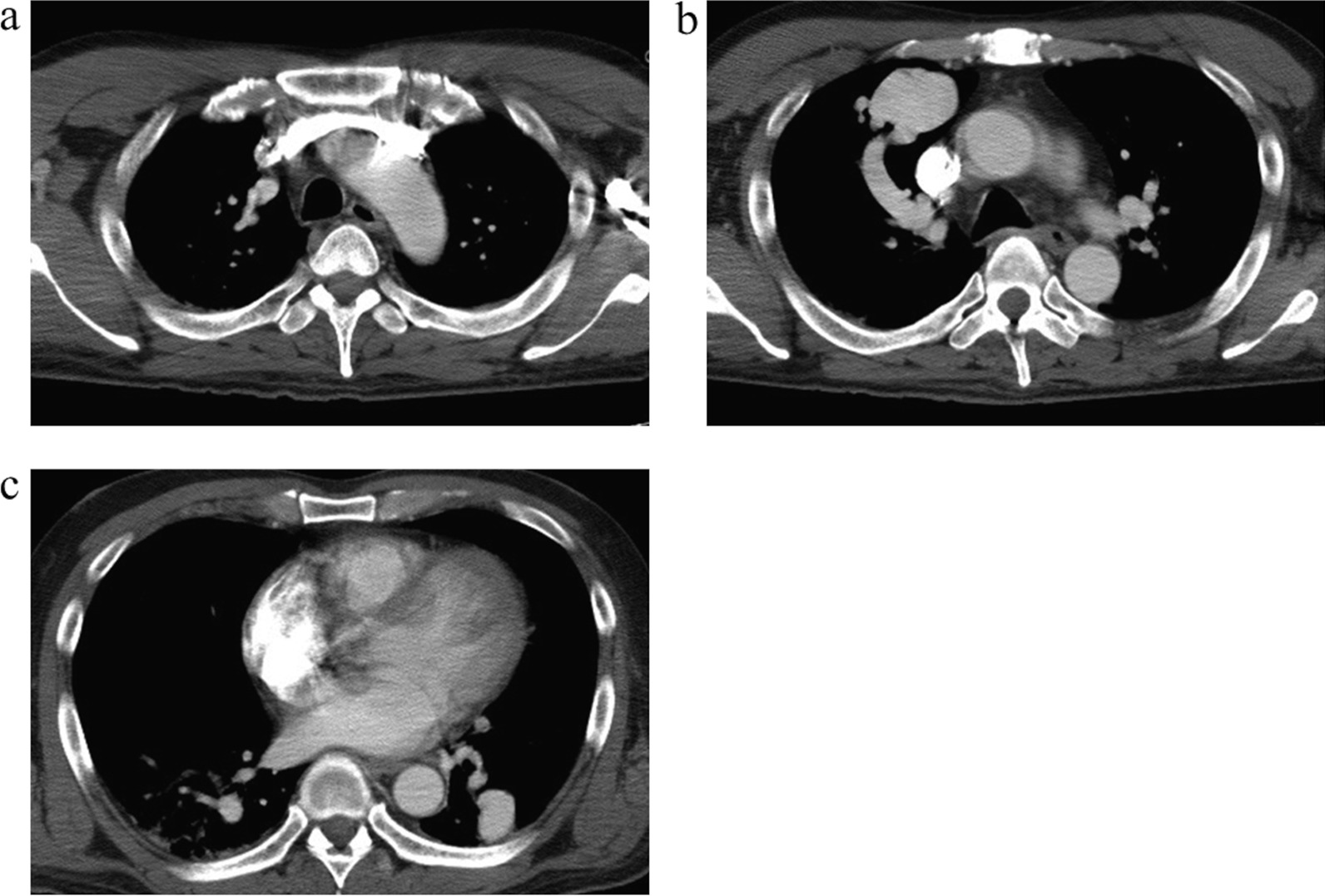
Enhanced chest computed tomography showed nodular lesions in right S1 (**a**), S3 (**b**) and left S10 (**c**)

A 51-year-old man presented to the emergency department of our institution with fever, consciousness disorder, and hypoxemia. Magnetic resonance imaging of the brain showed multiple ring-enhancing lesions, which suggested multiple brain abscesses (Fig. 1). Chest roentgenography revealed an abnormal shadow, and contrast computed tomography showed bilateral PAVMs: a 21 × 13 mm nodule in the right S1, preceded by a dilated pulmonary artery of 1 mm in diameter, a 46 × 34 mm nodule in the right S3, preceded by a dilated pulmonary artery of 11 mm in diameter, and a 28 × 25 mm nodule in the left S10, preceded by a dilated pulmonary artery of 1 mm in diameter (Fig. 2). The transtricuspid pressure gradient was 8.8 mmHg on the basis of the results of the echocardiogram, which did not indicate the presence of pulmonary hypertension. According to the medical consultation after improving the general condition, the patient had been diagnosed with PAVM using chest roentgenography and computed tomography in a medical checkup. In addition, the patient was suffering from exertional dyspnea for a long time period, but he has not received either any follow-up examinations or treatments at his own discretion. Because of the family history of PAVM, the patient’s history of epistaxis, and the existence of oral mucosa telangiectasia on a physical examination, in addition to PAVMs, he was diagnosed with HHT [4], and the brain abscess was thought to be caused by intrapulmonary right-to-left shunt.

Thanks to the intravenous administration of antibiotics, the brain abscess resolved, and the neurological symptoms improved; however, his hypoxemia was not ameliorated. Because pneumonia also occurred during the treatment of the brain abscess, the patient was intubated and an artificial respirator was used. Despite positive pressure ventilation, his hypoxemia worsened. During pressure-controlled ventilation with driving pressure at 14 cmH_2_O and PEEP at 6 cmH_2_O, breathing 100% oxygen, an arterial blood gas analysis revealed a partial pressure of arterial oxygen (PaO_2_) of 48.8 mmHg. However, changing the ventilator settings (driving pressure at 10 cmH_2_O and PEEP at 0 cmH_2_O) improved his oxygenation: PaO_2_ of 62.8 mmHg while breathing 100% oxygen. We suspected this was because positive pressure ventilation exacerbated the intrapulmonary right-to-left shunt.

Even after the improvement of pneumonia with the use of additional antibiotics and ventilator weaning, an arterial blood gas analysis revealed PaO_2_ of 58.7 mmHg while breathing 70% oxygen. In order to improve his hypoxemia and prevent a relapse of the neurological complications, we planned to treat his PAVMs. We considered that surgical treatment was suitable for the large PAVM in the right S3, due to the safety and certainty of therapy; however, the difficulty in maintaining stable breathing during positive pressure and one-lung ventilation initially prevented us from selecting surgical treatment. Because of the control of the patient’s infectious diseases, transcatheter embolotherapy was performed to treat the left PAVM.

**Fig. 3:**
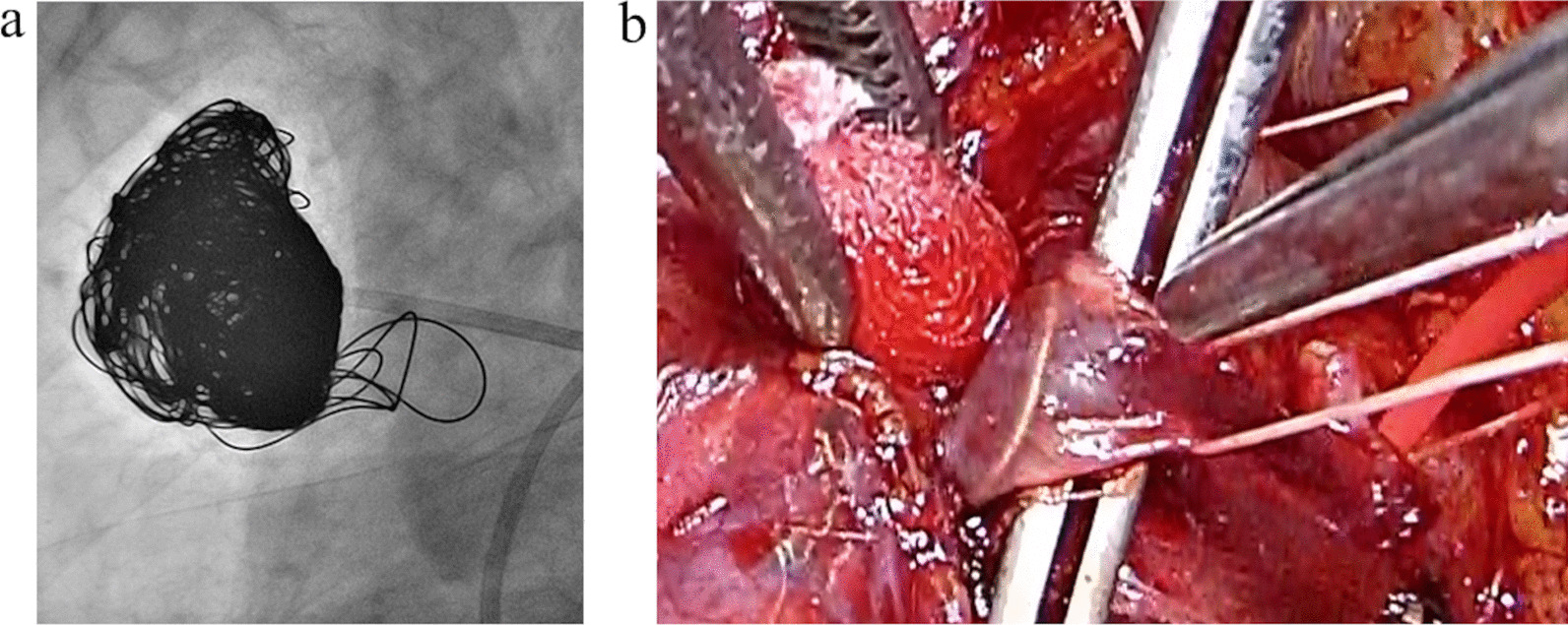
Arteriography after embolization revealed absence of blood flow in pulmonary arteriovenous malformation in right S3 (**a**). We confirmed the deviation of coil in the draining vein (**b**)

The occlusive coils embolized the left PAVM, which was located in S10, resulting in a slight improvement of the patient’s hypoxemia. After embolizing the largest PAVM on the right, which was located at S3, the patient’s oxygenation improved (Fig. 3a). Some occlusive coils protruded from the PAVM in the S3 and reached the V3; however, these coils were so poor in mobility that we left them untouched. After embolotherapy, an arterial blood gas analysis revealed that the PaO_2_ had improved to 82.7 mmHg while breathing room air. The patient was able to undergo rehabilitation.

Surgical treatment was performed under one-lung ventilation in the left lateral decubitus position. A 20-cm anterolateral skin incision was made along the fourth intercostal space. We confirmed the PAVMs of S1 and S3 and performed right upper lobectomy. In surgery, we found a coil was present in V3 after dissection of the mediastinal pleura and connective tissue. We therefore transected V1, V2, and V3, to avoid cutting or releasing the coil (Fig. 3b). After the transection of V1, V3, and A1 + 3, the patient’s oxygenation showed a remarkable improvement.

Transcatheter embolotherapy and surgical treatment were uncomplicated. The patient’s PaO_2_ level while breathing 100% oxygen increased up to 452 mmHg, and his dyspnea was ameliorated. Using pulmonary shunt fraction measurement with the 100% oxygen method, the shunt fraction was found to have improved from 27.9 to 11.8%. He was discharged to home on the 37th postoperative day after rehabilitation.

## Discussion

Patients with PAVM can present hypoxemia due to anatomic right-to-left shunt. Since PAVMs may cause life-threatening complications such as ischemic strokes, cerebral or peripheral abscesses, hemoptysis, and hemothorax, PAVMs of any size should be considered treatment regardless of its size and complications [5]. Because of concern about the recurrence of neurological complications, we aimed to control the patient’s PAVMs immediately. However, an examination was required to select the therapeutic strategy, considering the decline in exercise capacity caused by the brain abscess and pneumonia. Treatment of PAVM includes surgical resection and transcatheter embolotherapy. Transcatheter embolotherapy is less invasive and able to preserve the lung function. This treatment could be performed for bilateral or multiple PAVMs; however, it might have been associated with a relatively high recurrence rate and involve risks, such as rupture of a fistula, perforation by the catheter, or embolism of systemic circulation [6, 7]. On the other hand, surgical resection could be more invasive due to the use of general anesthesia, resulting in greater damage to the lung in comparison to transcatheter therapy. However, surgery would offer a chance of curative treatment of the patient’s PAVM, irrespective of the size [8]. Because of the decline in exercise capacity, in addition to the existence of bilateral PAVMs, transcatheter embolization, which is a relatively less invasive treatment was considered to be a first choice. However, we judged that the PAVM in the right S3 was so large that surgical resection would be more desirable from the perspective of curability. It is noteworthy that distention in the pulmonary vascular bed caused by positive pressure ventilation could increase the pulmonary vascular resistance and redistribute blood flow in the PAVM, where vascular resistance is relatively small, resulting in the exacerbation of intrapulmonary right-to-left shunt [9–11]. We therefore considered that maintaining stable breathing during positive pressure and one-lung ventilation would be difficult under general anesthesia. Because the patient’s infectious diseases were under control, we subsequently dealt with the PAVM in the left lower lobe using transcatheter embolization. In addition, we were able to confirm that embolization of the PAVM in the right S3 reduced the rate of blood flow with intrapulmonary right-to-left shunt and contributed to improving the patient’s oxygenation. Transcatheter embolization alone was not sufficient to curatively treat the patient’s PAVMs; however, it increased the patient’s exercise capacity, enabled him to undergo harder rehabilitation, and facilitated the performance of surgery under general anesthesia. These treatments allowed us to safely perform surgical resection of the PAVMs in the right upper lobe, and allowed the patient to return to his previous life—in fact, he was more comfortable than before treatment. There have been several reports about therapeutic strategies for multiple PAVMs, including surgery and endovascular therapy [6, 12]; however, combined treatment might be an option. Taken together, the adaptation and combination of therapies, as well as the sequence of treatments, should be considered depending on the patient status and lesions.

## Conclusion

Treatment for PAVM includes surgical resection and transcatheter embolotherapy. We successfully treated a patient with bilateral PAVMs in bad condition using a combination of these treatments. Appropriate treatments should be selected depending on the number and size of lesions and the patient’s physical status.

## Data Availability

Please contact author for data requests.

## Abbreviations

PaO_2_: Partial pressure of arterial oxygen
PAVM: Pulmonary arteriovenous malformation
HHT: Hereditary hemorrhagic telangiectasia
